# IMPACT OF ALZHEIMER’S DISEASE ON SUPRACHIASMATIC NUCLEUS CONNECTIVITY, SLEEP REGULATION, AND CIRCADIAN RHYTHM

**DOI:** 10.1093/geroni/igae098.2306

**Published:** 2024-12-31

**Authors:** Yiyi Shen, Hugo Calligaro, Michael Lam, Brian Khov, Keunyoung Kim, Wonkyu Ju, Mark Ellisman, Satchidananda Panda

**Affiliations:** Salk Institute for Biological Studies, San Diego, California, United States; Salk Institute for Biological Studies, San Diego, California, United States; University of California San Diego, La Jolla, California, United States; University of North Carolina, Chapel Hill, North Carolina, United States; University of California San Diego School of Medicine, San Diego, California, United States; University of California San Diego School of Medicine, San Diego, California, United States; University of California San Diego School of Medicine, San Diego, California, United States; Salk Institute for Biological Studies, San Diego, California, United States

## Abstract

Circadian disruption, notably sleep disturbances, serves as an early indicator of Alzheimer’s disease (AD), preceding cognitive symptoms such as memory impairment. Despite this, the underlying anatomical and functional mechanisms driving circadian disruption in AD remain elusive. The suprachiasmatic nucleus (SCN) serves as the central pacemaker regulating circadian rhythm and as the principal hub for entraining circadian rhythm. Here, we explore the relationships between SCN connectome evolution, sleep regulation, and circadian rhythm disruption in the APP/PS1 mouse model. In 3 to 8-month-old APP/PS1 mice, we obtained SCN image volumes via serial block-face scanning electron microscopy. We noted serval modifications in SCN connectomics, including a dendro-dendritic chemical synapse network reduction, crucial for synchronicity between SCN neurons, and a reduction of axonal boutons volume. In addition, we recorded electroencephalograms, electromyograms, temperature, and locomotor activity of 8-month-old WT and APP/PS1 mice to assess their sleep patterns. APP/PS1 mice displayed significantly reduced rapid-eye-movement sleep, associated with a reduced daily temperature amplitude and locomotor hyperactivity. Lastly, in 10 to 15-month-old APP/PS1 mice, we measure locomotor and metabolic activity change in response to acute light stimulation and light-dark cycle shift. APP/PS1 mice showed a decreased circadian fluctuation in metabolic activity and an impaired response to acute light pulse stimulation or phase shift. These preliminary results suggest considerable changes in the SCN connectivity may be responsible for circadian disruption in those with AD. Further investigation will evaluate the use of non-pharmacological approaches to mitigate the observed circadian disruption.

